# Mapping patient pathways and understanding clinical decision-making in dengue management to inform the development of digital health tools

**DOI:** 10.1186/s12911-023-02116-4

**Published:** 2023-02-02

**Authors:** Quang Huy Nguyen, Damien K. Ming, An Phuoc Luu, Ho Quang Chanh, Dong Thi Hoai Tam, Nguyen Thanh Truong, Vo Xuan Huy, Bernard Hernandez, Jennifer Ilo Van Nuil, Chris Paton, Pantelis Georgiou, Nguyet Minh Nguyen, Alison Holmes, Phan Vinh Tho, Sophie Yacoub, the Vietnam ICU Translational Applications Laboratory (VITAL) investigators

**Affiliations:** 1 Centre for Tropical Medicine, Oxford University Clinical Research Unit, Ho Chi Minh City, Vietnam; 2 Centre for Antimicrobial Optimisation (CAMO), Imperial College London, London, UK; 3 Hospital for Tropical Diseases, Ho Chi Minh City, Vietnam; 4 Centre for BioInspired Technology, Imperial College London, London, UK; 5 Department of Information Science, University of Otago, Dunedin, New Zealand; 6 Centre for Tropical Medicine and Global Health, Nuffield Department of Medicine, University of Oxford, Oxford, UK

**Keywords:** Dengue, Clinical decision-making, Decision support, Digital health, Implementation research, Vietnam

## Abstract

**Background:**

Dengue is a common viral illness and severe disease results in life-threatening complications. Healthcare services in low- and middle-income countries treat the majority of dengue cases worldwide. However, the clinical decision-making processes which result in effective treatment are poorly characterised within this setting. In order to improve clinical care through interventions relating to digital clinical decision-support systems (CDSS), we set out to establish a framework for clinical decision-making in dengue management to inform implementation.

**Methods:**

We utilised process mapping and task analysis methods to characterise existing dengue management at the Hospital for Tropical Diseases, Ho Chi Minh City, Vietnam. This is a tertiary referral hospital which manages approximately 30,000 patients with dengue each year, accepting referrals from Ho Chi Minh city and the surrounding catchment area. Initial findings were expanded through semi-structured interviews with clinicians in order to understand clinical reasoning and cognitive factors in detail. A grounded theory was used for coding and emergent themes were developed through iterative discussions with clinician-researchers.

**Results:**

Key clinical decision-making points were identified: (i) at the initial patient evaluation for dengue diagnosis to decide on hospital admission and the provision of fluid/blood product therapy, (ii) in those patients who develop severe disease or other complications, (iii) at the point of recurrent shock in balancing the need for fluid therapy with complications of volume overload. From interviews the following themes were identified: prioritising clinical diagnosis and evaluation over existing diagnostics, the role of dengue guidelines published by the Ministry of Health, the impact of seasonality and caseload on decision-making strategies, and the potential role of digital decision-support and disease scoring tools.

**Conclusions:**

The study highlights the contemporary priorities in delivering clinical care to patients with dengue in an endemic setting. Key decision-making processes and the sources of information that were of the greatest utility were identified. These findings serve as a foundation for future clinical interventions and improvements in healthcare. Understanding the decision-making process in greater detail also allows for development and implementation of CDSS which are suited to the local context.

**Supplementary Information:**

The online version contains supplementary material available at 10.1186/s12911-023-02116-4.

## Background

Dengue is an infection of global public health importance. The disease exerts a significant burden on healthcare systems, particularly during seasonal epidemics when hospitalisations increase significantly. Infection can result in life-threatening complications including severe bleeding, hypovolaemic shock and organ failure [1]. The backdrop of human-induced climate change, complex interactions in human behaviour including travel and mobilisation, *Aedes* mosquito vector and the physical environment have resulted in a rapidly-expanding worldwide distribution of disease [2, 3].

Clinical management for severe dengue is complex. Treatment focuses on restoring effective circulating volume balanced with avoiding fluid overload. Other complications of the disease include profound bleeding and organ dysfunction, both requiring specialised management and monitoring in hospital settings. It is essential—yet challenging—to identify the patients who are at high risk of progression to severe disease during early illness. To assist clinicians in dengue management, national and international guidelines for dengue clinical management have been developed [4, 5]. Research on the formulation of clinical risk scores [6], and more recently models using machine learning [7] have proliferated as a means to provide decision support for clinicians. Optimising healthcare decisions at different stages of patient presentation offers multiple benefits, including improving outcomes and efficiency in resource use, which can often be limited within endemic settings.

Ensuring the effective implementation of these adjunctive tools, termed clinical decision support systems (CDSS), can be fundamentally challenging for different reasons. Real-world performance of interventions vary significantly and are in part affected by the heterogeneity in health services, the nature of dengue management [8], as well as intrinsic complexities involved in the implementation of such support systems [9]. The impact of differences in care priorities, staff training, healthcare pathways, resource availability and poor understanding of context mean that many top-down approaches to improve healthcare suffer from a lack of external validity. For example, in an cluster randomised study examining use of insecticide treated nets in real-world settings, individuals in clusters assigned to the intervention were paradoxically at higher risk of dengue, at odds with previous research data [10]. Health systems research has consistently demonstrated the importance of understanding the local environment and processes [11] to lead to effective implementation, yet there exists a paucity of work in this area.

As a part of research into the clinical management of dengue, our team is developing digital health tools that are data-driven and focused on providing clinical decision-support [12]. These tools can be used at the point of patient contact to provide information, which would result in clinical decisions of better, and/or more consistent quality with the aim of improving patient outcomes.

To understand how these tools could be applied to maximise uptake and utility, we carried out a study mapping existing healthcare processes, clinical reasoning and cognitive factors to establish a framework for decision-making in dengue. The objectives of the study were to understand how clinicians and potential CDSS end-users deliver care to dengue patients, and to set up a baseline to facilitate the future implementation of the digital tools under development.

## Methods

### Setting

The research was carried out at the Hospital for Tropical Disease (HTD) in Ho Chi Minh City, Vietnam. HTD is a 700-bed government specialist hospital, which provides specialist care facilities for infectious diseases, including emergency resuscitation, inpatient and outpatient treatment, and adult and paediatric intensive care facilities. The hospital is located in the city and accepts referrals from self-presenting patients and other healthcare facilities in the Ho Chi Minh City and Southern Vietnam catchment area, which has a combined population of approximately 40 million people. The facility manages around 30,000 patients with clinically diagnosed dengue each year, of which 8000–15,000 are admitted for dengue-specific treatment. The study was carried out between September 2020 and March 2022, before and during COVID-19 controlling measures were implemented at the hospital.

### Approaches

Methods used included a ‘last 10 patients’ approach [13] to initially describe the existing delivery of care for adults and children with dengue. An ethnographic approach was used subsequently as adjunctive methodology to understand treatment pathways of dengue patients at the HTD [14]. This was done through direct observation of team dynamics and patient management by a clinician-researcher (NQH) who was embedded in the clinical teams, but did not take part in patient care. The treatment pathways were described from the first day of patient arrival at the outpatient department with clinically suspected dengue until the day they fully recovered. The sampling frame of observation included activities carried out by healthcare staff in dengue management within the emergency department, outpatient units, adult and paediatric intensive care units, and inpatient wards. Direct observations by the clinician-researcher were recorded in fieldnotes and key areas were followed up with informal discussions with the individual care providers.

Second, we conducted a task analysis of primary stakeholders in order to characterise the processes involved throughout the pathway [15]. The stakeholders were medical staff of the HTD who were directly, or indirectly involved in the dengue management pathway. The primary stakeholders included: clinicians, nurses, nursing assistants, administrative nurses and auxiliary healthcare staff. Information including timeframes, use of infrastructure, adjuncts, roles and responsibilities of each of the stakeholders identified was captured for the same consecutive series of patients as above who were admitted with dengue.

For a greater in-depth characterisation of decision-making, we conducted semi-structured interviews with a subset of the primary stakeholders. We selected different groups of clinicians from those included in the process mapping. The research group developed the interview guides based on the findings from the dengue care pathway and task analysis results. These interviews underwent iterative development and were piloted on clinicians within the group. A list of interview question stems is shown in Table 1.

**Table 1:** Interview stems for semi-structured interviews with clinicians, conducted online in Vietnamese over 45 min

List of interview stems used for semi-structured interviews
(1) Remember a case of dengue you have seen recently – what do you consider as the most important clinical decisions to make in your role?
(2) How do you tell dengue apart from other infectious diseases which cause fever? Is this difficult, what things do you find helpful in your practice?
(3) Describe how you would assess the severity of a patient with dengue
(4) How do you know or decide when a patient should be admitted, discharged or referred to another service (ICU or ward)?
(5) How do you define a complex dengue case?
(6) How do you assess if a patient needs intravenous fluids or blood therapy? What information do you base this decision on?
(7) How do final decisions about management in a patient with dengue get made?
(8) How do you feel if you had to manage cases of dengue outside the age group you are used to? How much difference is there in your management between an adult or a child?
(9) Can you tell me what personal or hospital factors allow for an ideal approach in dengue management?
(10) If a computer program could take in clinical and laboratory information and accurately provide a risk of severe dengue for a patient, would it be used, and how?

We focused specifically on the role of doctors as they were responsible for the majority of clinical decision-making for dengue at the hospital. We used purposive sampling to gain a wide range of perspectives and experiences. Respondents were identified through hospital peer networks and nominated by respective heads of department. Of all respondents who participated in the interviews, half were based in intensive care units with another half from ward and outpatient-based settings (Table 2).

**Table 2:** Demographic characteristics of interview respondents

Characteristic of interviewed clinicians	N (%)
Years of experience IQR range, median	3–12, 8
Ward	
Adult intensive care unit	3 (30)
Pediatric intensive care unit	2 (20)
General adult	1 (10)
General pediatric	4 (40)

Interviews were carried out in Vietnamese by researchers trained in qualitative interviewing, accompanied by a clinician researcher (NQH). Due to COVID-19 restrictions all interviews were performed virtually using video-conferencing software. We audio-recorded the interviews, with the explicit consent from the participants. A third party outside the research team transcribed the interviews into Vietnamese and then translated it into English. Interviews were continued until thematic saturation was reached, which we assumed would be between 8 and 12 interviews.

### Data analyses

Analysis for data collected from the task analysis was done through decomposition of activities into their fundamental tasks, describing the technical environment including location, devices, method and the typical durations of each event. Coding of the interview findings from transcripts was done independently by three researchers (NQH, LPA, DM) using a grounded theory approach [16]. Open coding was done independently and the aggregation of codes were done through axial coding and team discussions. These codes were subsequently presented to the wider dengue research group through meetings. An iterative process of synthesis was done in order to derive the higher-level emergent themes presented [17].

## Results

### Patient pathway and clinical processes

**Fig. 1:**
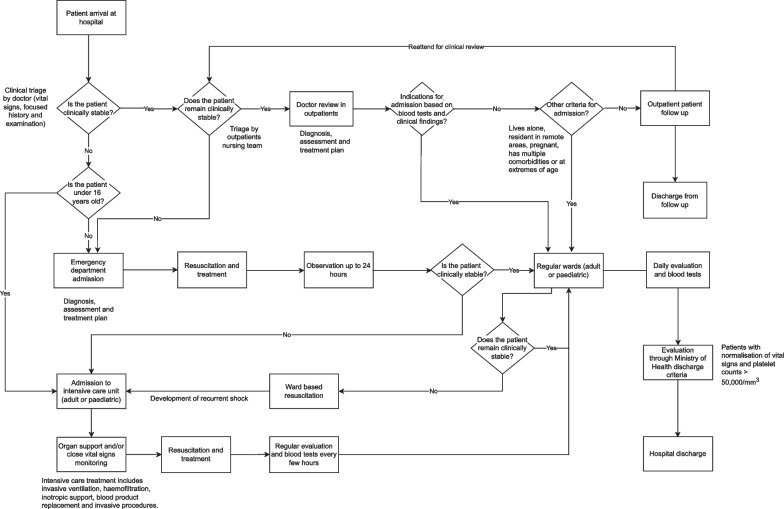
Process map of dengue care at the Hospital for Tropical Diseases, Vietnam constructed from patient observations

A process map of patient management and movement between departments at the HTD was constructed to provide contextual background (Fig. 1). Ten consecutive patients including adults and children with a confirmed diagnosis of dengue were observed throughout their stay from hospital presentation to discharge. These patients had a median length of stay of 6 days (IQR 3–10).

Key decision-making steps in the pathway were identified as follows:

- Patients were triaged on hospital arrival and managed according to age, clinical severity and risk factors (obesity, pregnancy, co-morbidities, etc.) according to guidelines.
- The majority of patients presenting to the hospital would then be managed on an outpatient basis with daily follow-up. Those with severe and/or unstable disease or with other risk factors would be admitted to adult or paediatric wards via the Emergency department or directly to intensive care units.
- The nature of dengue and lack of tools to accurately stratify disease progression risk meant that frequent clinical assessments were required, and treatment pathways between settings often crossed over resulting in redundant system but also some duplication of work.
- We observed that the Ministry of Health (MOH) dengue guidelines played a significant role in the management of dengue cases, especially during the early stages of presentation.

Clinical decision-making and triage throughout these processes was predominantly doctor-led. Supervision was provided by senior staff from the formalised hospital structure where explicit approval was required for treatment in difficult cases.

Using task analysis methods we characterised individual processes of each healthcare role, capturing the technical environment, location and typical durations of their shift? in detail. An overview of the care pathways for adults and paediatric patients is shown in Fig. 1 and the results of task analysis are shown in the Additional file 1: Appendix.

### Clinical decision-making results

To characterise and understand the decision-making processes involved during patient management, we performed semi-structured interviews between 20th September 2021 and 6th January 2022 with hospital doctors directly involved in the care of dengue patients. Thematic saturation was achieved after 10 interviews. Respondents worked in adult intensive care unit (n = 3), paediatric intensive care unit (n = 2), general paediatrics (n = 4) and adult medicine wards (n = 1). The median years of experience was 8 (IQR–12 years). We identified the following themes:

1. Information from clinical evaluation is prioritised and is the main driver in early dengue decision-making.

Respondents consistently emphasized the role of clinical evaluation such as history taking and risk assessment based on information from vital signs, examination findings and epidemiological factors to differentiate dengue from other infectious diseases:

‘…clinical speaking, the presentation of dengue fever is similar that of other viral infections. A patient with dengue normally has a high temperature which is very prolonged, and might be initially responsive to antipyretics but will later experience fever again. Management depends on their clinical manifestations, location of care and seasonal factors.’ – Resident in medicine, 3 years experience.

Experienced clinicians also expressed the importance of their knowledge of clinical signs and use of vital signs measurements in differentiating dengue from complex bacterial infections:

‘For patients with septic shock, the first thing that we observe is their pronounced lethargy, and other signs such as having cool arms and legs, blue lips or other related symptoms. Generally speaking, it is predictable whether a patient is experiencing septic shock or shock resulting from dengue fever based on their clinical symptoms…’—Consultant in medicine, 11 years experience.

‘In patients with septic shock, their blood pressure would normally drop more dramatically…if we [are] observant, we can figure it (the diagnosis) out.’—Consultant in intensive care, 9 years experience.

The timing of illness onset is consistently used an important parameter to predict onset of the critical phase and is used to guide the intervention and monitoring:

‘First things first, it’s the day of illness. Usually, a case of dengue may turn critical between days four and seven and these patients may require extra attention.’ – Consultant in paediatrics, 20 years experience.

However, other non-clinical risk factors may also influence need for hospitalisation:

‘There are some cases that I request to be hospitalized. For example, those patients from somewhere far away, or those who live alone at home with no one to take care of them.’ – Resident in medicine, 3 years experience.

2. Dengue-specific diagnostic tests and non-specific tests are useful in dengue patient management but have limitations

Rapid diagnostics such as the non-structural protein 1 (NS1) lateral flow assay and nucleic acid amplification tests (NAAT) are available for dengue diagnosis in hospital and clinic settings. In early disease state, NS1 is useful in differentiating Dengue and other infectious disease.

'…so during the first three days of illness…. we have tests which can be used for diagnosis such as NS1 or NAAT.’ – multiple respondents.

Occasionally, the long turnaround time for NS1 results and the limited availability of NAAT tests affected decision-making. Other laboratory tests utilized for clinical decision-making included blood haematocrit and inflammatory markers such C-reactive protein (CRP) test. These are often used to differentiate between dengue and potential bacterial infections and guided patient management.

‘…NS1 results may take a while to return, possibly take hours, whilst we need the results of clinical investigation immediately. At times we might not obtain a result until the following day so we rely on rapid tests such as the blood haematocrit, combined with information on epidemiology and clinical signs…’ – Consultant in intensive care, 20 years experience.

‘…if no source of infection is identified in the patient and their CRP values are not high, it must be a case of dengue fever and not a bacterial infection.’ – Resident in paediatrics, 7 years experience.

‘Although a patient with dengue may not exhibit critical signs of illness, a low value of platelet count may be sufficient to admit them to hospital.’ – Consultant in medicine, 11 years experience.

3. Dengue management is protocolised by guidelines but complexities arise in later stages of the disease

All respondents refer to the Vietnamese Ministry of Health (MOH) national dengue guidelines to guide management strategies for patients in the early stage (days 3 or 4) of illness. The guidelines include explicit criteria for admission and algorithms for supportive fluid treatment. However, treatment decisions during and after the critical phase of the disease or in specific populations may be deferred to more senior clinical staff.

'… in patients where the protocol is applied but no responsiveness is seen, some experienced doctors who are considered experts may try a small customization in treatment. This is done based on clinical experience and not part of the hospital's protocol.' – Resident in medicine, 3 years experience.

A common example not covered in the guidelines is recurrent dengue shock, where patients develop a second or multiple episodes of shock after initial fluid resuscitation and stabilization.

'…but when a patient has repeated episodes of shocks up to three, four, or five times and this is not included in the guidelines, we have to rely on the patient's individual clinical state in order to figure out the appropriate treatment.’ – Consultant in intensive care, 9 years experience.

Following guidelines is essential in treatment delivery, but at times their interpretation can be challenging. Respondents describe the lack of a formalised system in order to assess illness severity and treatment response.

‘In the later stage of disease, we don’t have any scoring scale for dengue patients. There is no system for the evaluation of a patient’s illness severity. Instead, doctors perform examinations and provide their expert opinion on the patient clinical state and prognosis…’ – Intern in medicine, 1 year experience.

4. External factors play a crucial role in clinical decision- making

The peak period of dengue season and the status of the hospital capacity may be used to adjust individual approaches to patient treatment. Respondents described the challenges in prioritising individual patient management whilst taking into account the wider needs of the community by effective utilisation of hospital capacity:

‘For example, when an epidemic comes - the hospital may become too occupied and there may be no beds available anymore. So sometimes patients have to lie on the floor, which is not comfortable. The staff get overloaded (with work), and they might not be able to complete all their tasks. At those times, I must consider whether to request daily check-ups on a patient as an outpatient, or advise them to go to other hospitals for care…’ – Resident in paediatrics, 4 years experience.

The opinion of the care-giver or relative of the patient is also an important factor in decision-making:

‘Examination and treatment must be done according to the wishes of a patient. But at times, the relatives might not agree and they might request for a hospital transfer or other aspects of care. During this time the doctors must also take into account the relative’s opinion and plan the patient management accordingly…’ – Consultant in paediatrics, 7 years experience.

Additionally, training courses on dengue management are useful when it comes to updating treating clinicians about new guidelines.

‘…Every year, the hospital provides training opportunities as dengue is a kind of annual epidemic, so they have repeated, especially when there is change or updates in the protocols, there is a training workshop for consolidation.” – Consultant in medicine, 11 years experience.

5. The implementation of digital clinical decision-support systems is welcomed, but important barriers need to be addressed.

Respondents had a positive outlook regarding the future role of a digital CDSS for their setting in Vietnam:

‘A computer system which can produce an explicit recommendation for approaching a problem will be the most helpful. It should be able to tell me how to monitor a patient, how often I should take tests, and how frequently to do this…’ – Resident in paediatrics, 3 years experience.

However, issues of interpretability of this system, governance by regulatory authorities, accountability of decisions, time efficiency, and the adverse impact on deskilling medical staff were raised as possible issues to implementation.

‘… if I use the system and the results conflict with my decision then I will have to review the patient again to see if I missed anything, as an individual can be wrong and make mistakes. But I have to understand the results of the system as I may be asked ‘why should you believe it?’ by my seniors and I won’t be able to explain this.’ – Resident in paediatrics, 7 years experience.

‘If the CDSS is used in peak dengue season when there are too many patients, we will not be able to use the system if it is onerous. We prefer to be able to read test results quickly but not spend time inputting data into the system….’ – Consultant in medicine, 11 years experience.

‘…, if someone is reliant on a software product to guide clinical management, it is possible that they might eventually lose their clinical sense… some clinicians may not accept the CDSS and rather prefer to rely on their knowledge.’ – Consultant in intensive care, 9 years experience.

## Discussion

Understanding context is crucial for translating research into real world settings [18]. Healthcare interventions such as decision-support systems should ideally address the needs, workflow and settings of end-users and patients in order to enhance adoption and sustainability [19]. As part of a multi-disciplinary project to improve clinical management of dengue through novel technological innovations (vital.oucru.org), we undertook this study to characterise the existing decision-making processes within our setting – a tertiary referral hospital in Vietnam.

Although there have been numerous studies published which focus on the development of predictive models for dengue clinical management – to our knowledge and through literature search there have been no studies which directly address the cognitive processes involved for decision-making at the individual level. Such models or systems developed in isolation are unlikely to result in clinical impact if implemented in such a way which do not formally consider local factors. Through mapping patient care pathways and interviews with clinicians, we describe the behaviour, reasoning and priorities in information utilisation employed by clinicians in delivering dengue patient care.

All respondents emphasised the importance of clinical assessment (i.e. history, examination and use of vital signs) in patient management. Priorities during the initial patient evaluation included differentiating dengue from other acute infections, and safe triage of patients according to their risk of deterioration. Guidelines adopted for routine clinical use include those published by the Vietnamese MOH and the WHO. Both set of guidelines utilise a checklist for identifying clinical ‘warning signs’, and vulnerable populations as criteria for patient hospital evaluation and treatment.

Although use of these guidelines can identify patients who are unlikely to experience severe disease effectively, they may result in otherwise unnecessary hospital admissions. This subsequently places additional strain on healthcare resources [20]. Processes involved in clinical evaluation are inherently subjective between practitioners, and dependent on factors such as training, clinical experience and clinical setting [21]. We performed this study at a tertiary infectious diseases hospital where clinicians are experienced in the management of dengue patients, but it is likely that the performance of such evaluations differ in other community or regional healthcare settings.

The NS1 lateral flow assay is suitable for near-patient testing but the workflow at our hospital meant that testing was performed in the laboratory. Although this ensures the quality of the testing process, it typically increases the result turnaround time. NS1 testing is also available outside of hospital settings through independent private laboratories. The result turnaround time for testing outside the hospital also tends to be shorter due to shorter queues but has a higher cost. Limitations in NS1 performance for patients with late disease were identified as a barrier by respondents to its use in decision-making. These findings illustrate a diagnostic gap – an important issue in low- and middle-income countries [22]. Laboratory investigations such as nucleic acid amplification (NAAT) tests provide a much higher performance for diagnosis in dengue. At our institution, the reference standard diagnostic test is through a multiplex real-time Reverse Transcriptase-Polymerase Chain Reaction (RT-PCR) method. However, RT-PCR is not utilised routinely for early dengue diagnosis as it has a significantly longer result turnaround time and the cost is prohibitive for most patients attending clinic. Recent developments which in part incorporate novel technologies in pathogen detection might be promising [23]. Roll out of rapid diagnostics, strengthening laboratory, medicine access and connectivity could lead to significant improvements in care [24], although actual clinical utility will depend on the mode of implementation [25].

In our interview responses clinicians have emphasised the importance of accessing contemporaneous vital signs information. Access to up to date information however can be a challenge in these settings [26]. The role of low-cost systems such as medical wearables have potential in bridging this gap by providing patient health information [27]. In our study we did not cover the issue of direct costs and patient out-of-pocket expenses. However this is likely to be an important factor in decision-making as novel, technological innovations are introduced over time [28, 29].

Much of clinical management in dengue is affected by seasonality. A dramatic increase in symptomatic dengue cases during the wet season results in a reorganisation of decision-making and allocation of hospital services [30]. Utilising knowledge of external factors including local epidemiology, disease outbreaks and bed capacity to inform clinical decision-making is particularly important. For decision-support, digital health technologies which can collate climatic data and local data in order to predict disease hold promise for a role in decision-support [31]. There is general optimism in the role of digital technology in Vietnam by clinicians and the public [32, 33], but issues relating to interpretability, trust and accountability as expressed by respondents need to be understood and addressed [34].

Strengths of the study include the involvement of clinicians with different levels of healthcare experience across different departments for process mapping and interviews. The ethnographic approach which involved observations over several weeks also adds to the validity of findings. Semi-structured interviews were designed in collaboration with the wider dengue research group, social sciences group, and clinical team. We used a grounded theory approach to analysis without use of existing models of decision-making, in order to better understand our setting. Our findings will directly facilitate a human-centred design approach for the development and evaluation of a CDSS in dengue. Although these findings are not directly transferrable to other settings and institutions, it is likely that the methodology utilised in this ‘bottom-up’ approach to development will be transferrable.

Limitations include study performed at a single centre, with focus was on healthcare clinical decision-making for only one disease only. The limited sample size of the interviewees also meant that these views might not be fully representative of the processes at other healthcare institutions in Vietnam. We did not employ mixed-methods approach to include quantitative data and this could have strengthened the findings in our study although this was outside the original intention of the study,

In conclusion, we have characterised pathways and decision-making processes in managing dengue in a low- and middle-income country tertiary hospital setting. Important issues relating to clinical priorities and use of information will directly inform the roles and scope of our CDSS development. The description of processes involved in dengue management will also serve as a foundation to inform other related healthcare interventions.

## Supplementary Information

**Additional file 1.** A table of roles and responsibilities mapped through task analysis for management of patients with dengue at the hospital.

## Data Availability

The datasets used and/or analysed during the current study available from the corresponding author on reasonable request.

## Abbreviations

CDSS: Clinical decision support system
CRP: C-reactive Protein
HTD: Hospital for Tropical Diseases
ICU: Intensive care unit
NS1: Non-structural protein 1
MOH: Ministry of Health
VITAL: Vietnam ICU Translational Applications Laboratory
