# Supplementary material for: Mapping patient pathways and understanding clinical decision-making in dengue management to inform the development of digital health tools
Source: BMC Med Inform Decis Mak. 2023 Feb 2;23:24. doi: 10.1186/s12911-023-02116-4 (PMC9893980; doi:10.1186/s12911-023-02116-4)
Supplement: Supplementary file 1 — Additional file 1. A table of roles and responsibilities mapped through task analysis for management of patients with dengue at the hospital. [file 12911_2023_2116_MOESM1_ESM.docx]

**Supplementary appendix**

Task analysis conducted through patient observation (n=10) over 3 weeks. Summary of of roles, responsibilties and the technical environment for each task done for dengue patient care.

| Role | Task activities observed | Technical environment | Duration of each event |
| --- | --- | --- | --- |
| Doctor | Completing patient admission proforma | Pen, paper, patient clinical notes | 5 minutes |
|  | Physical examination of patient | Patient bedside | 5 minutes |
|  | Updating orders and patient status on progress notes | Ward, pen, paper, patient clinical notes | 5 minutes |
|  | Obtaining consent for additional interventions | Discussion with patient or relatives, pen, consent form | 5 minutes |
|  | Set up of vital signs monitors | Vital signs monitors - wall mounted | 4 minutes |
|  | Recording patient summary on round | Patient clinical notes, verbal communication with lead doctor/ vice-lead doctor | 5-15 minutes |
|  | Update patient on electronic health record system | Desktop computer located on wards | 4 minutes |
|  | Placement of invasive central venous catheter | Patient bedside using central venous catheter, monitoring device and set up | 30 minutes |
|  | Completion of nutrition form | Nutrition evaluation, pen, patient clinical notes | 10 minutes |
|  | Consultation with senior / consultant clinician | Meeting over phone or through clinical rounds | 15 minutes |
|  | Manual monitoring of vital signs | Blood pressure monitor, watch, pulse oximeter | 2 minutes |
|  | Measurement of haematocrit using bedside testing | Bedside haematocrit testing equipment | 5 minutes |
|  | Patient communication | Patient bedside | 2 minutes |
|  | Complete discharge summary | Patient clinical notes and pen on the ward | 5 minutes |
|  | Clinical examination | Patient bedside | 5 minutes |
|  | Order laboratory testing | Patient clinical notes and complete request form | 5 minutes |
| Nurse | Measure vital signs | Blood pressure cuff, watch, pen, patient clinical notes | 3 minutes |
|  | Fill general evaluation form and estimate evaluation score | Evaluation form, pen | 10 minutes |
|  | Prepare medical equipment | Monitors, ventilator, closed suction catheter | 5 minutes |
|  | Prepare IV line for fluids administration | Intravenous fluids and giving set by patient bedside | 3-5 minutes |
|  | Filling daily care form | Pen, patient notes | 5 minutes |
|  | Assist patient getting dressed | Patient's clothes | 3 minutes |
|  | Transferring patient from outpatients/emergency department | Patient bed | 10 minutes |
|  | Communication with doctor | Phone, in person meeting | 1 minute |
|  | Patient hygiene | Water, cloth, cleaning | 10 minutes |
|  | Preparing IV fluids | Intravenous fluids and giving set | 5 minutes |
|  | Collect blood sample for CBC | Lab blood tubes, needle and syringe. | 2 minutes |
|  | Medical equipment preparation | Procedures trolley, central lines and catheters | 2 minutes |
|  | Filling general plan form | Patient clinical notes, pen | 5 minutes |
|  | Check identity bracelet | Patient bedside | 1 minute |
|  | Remove all IV lines | Patient bedside | 5 minutes |
|  | Patient discussion | Patient bedside | 3-5 minutes |
|  | Medical equipment replacement when needed | Medical equipment | 2-4 minutes |
|  | Filling evaluation form | Patient clinical notes, pen | 10 minutes |
| Nursing assistant | Move patient from OPD/ED to AICU | Movable beds | 10 minutes |
|  | Bring blankets, warm kettle | ICU blankets, kettle | 5 minutes |
|  | Support patient dressing | Patient gowns | 3 minutes |
|  | Bring lab samples to laboratory | Collected lab tubes, urine sample | 10 minutes |
|  | Support patient to get out from bed | ICU bed, wheel chair if required | 2 minutes |
|  | Clean the area after discharge | Cleaning equipment | 10 minutes |
|  | Bring fees to planning management department | Fees printed out from administrative nurse | 7 minutes |
|  | Take relatives to counter for payment for medical care | Payment counter | 10 minutes |
| Administrative nurse | Patient's insurance health check | Online system E-hospital using computer, discussion with relatives | 10-15 minutes |
|  | Calculate overall hospital fees | Online system E-hospital using computer | 10-15 minutes |
|  | Refund if necessary | Cash | 10 minutes |
|  | Check signatures of doctors | Patient clinical notes | 10 minutes |
|  | Check patient notes before discharge | Patient clinical notes | 10 minutes |
| Paramedic | Transfer patients between wards | Movable beds | 10 minutes |
|  | Maintain ward hygiene | Cleaning equipment | 30 minutes |
|  | Transfer clinical samples to laboratory | Sample container | 10 minutes |
|  | Collect testing results from laboratory | Results slip, patient clinical notes | 10 minutes |
